# Neurotensin Co-Expressed in Orexin-Producing Neurons in the Lateral Hypothalamus Plays an Important Role in Regulation of Sleep/Wakefulness States

**DOI:** 10.1371/journal.pone.0062391

**Published:** 2013-04-19

**Authors:** Naoki Furutani, Mari Hondo, Haruaki Kageyama, Natsuko Tsujino, Michihiro Mieda, Masashi Yanagisawa, Seiji Shioda, Takeshi Sakurai

**Affiliations:** 1 Department of Molecular Neuroscience and Integrative Physiology, Faculty of Medicine, Kanazawa University, Kanazawa, Ishikawa, Japan; 2 Department of Anatomy, Showa University School of Medicine, Tokyo, Japan; 3 Center for Behavioral Molecular Genetics, University of Tsukuba, Tsukuba, Japan; 4 International Institute for Integrative Sleep Medicine, University of Tsukuba, Tsukuba, Japan; 5 Howard Hughes Medical Institute, University of Texas Southwestern Medical Center, Dallas, Texas, United States of America; Tokyo Metropolitan Institute of Medical Science, Japan

## Abstract

Both orexin and neurotensin are expressed in the lateral hypothalamic area (LHA) and have been implicated in the regulation of feeding, motor activity and the reward system. A double label immunofluorescence and *in situ* hybridization studies showed that neurotensin colocalizes with orexin in neurons of the LHA. Pharmacological studies suggested that neurotensin excites orexin-producing neurons (orexin neurons) through activation of neurotensin receptor-2 (NTSR-2) and non-selective cation channels. *In situ* hybridization study showed that most orexin neurons express *neurotensin receptor-2* mRNA but not *neurotensin receptor-1* (*Ntsr-1*) mRNA. Immunohistochemical studies showed that neurotensin-immunoreactive fibers make appositions to orexin neurons. A neurotensin receptor antagonist decreased Fos expression in orexin neurons and wakefulness time in wild type mice when administered intraperitoneally. However, the antagonist did not evoke any effect on these parameters in orexin neuron-ablated mice. These observations suggest the importance of neurotensin in maintaining activity of orexin neurons. The evidence presented here expands our understanding of the regulatory mechanism of orexin neurons.

## Introduction

Neurotensin is a 13 amino-acid residue neuropeptide implicated in neuroendocrine function, feeding behavior and the reward system. Neurotensin acts on three subtypes of neurotensin receptors (NTSRs); NTSR-1, NTSR-2, and NTSR-3. NTSR-1 and NTSR-2 are G-protein coupled receptors, while NTSR-3 is a single transmembrane receptor [1], [2]. NTSR-1 couples to G_q_ proteins and activates phospholipase C (PLC) and inositol phosphate (IP) signaling [3], [4]. NTSR-2 shows lower affinity to neurotensin, and its physiological role remains unclear. Little is known about the physiological role of NTSR-3 [5].

Orexin A and orexin B (also known as hypocretin 1 and hypocretin 2) are neuropeptides that are expressed in a population of neurons in the lateral hypothalamic area (LHA) [6], [7]. Disruption of this neuropeptide system causes narcolepsy in human and animals, suggesting that orexins are crucial regulators of sleep and wakefulness [8]. Orexin-producing neurons (orexin neurons) send axonal projections widely in CNS regions that regulate emotion, sleep, wakefulness, arousal, the reward system and energy homeostasis [9]. Especially dense projections of orexin neurons are found in the monoaminergic and cholinergic nuclei in the hypothalamus and brainstem [10]–[15]. Orexin neurons regulate cholinergic/monoaminergic neurons to maintain a long, consolidated waking period. Orexin neurons receive abundant input from the limbic system, preoptic area, posterior hypothalamus and brain stem [16], [17].

Activity of orexin neurons is regulated by a number of factors, including cholecystokinin, neurotensin, vasopressin, oxytocin, neuropeptide Y (NPY), adenosine, serotonin, and noradrenaline [18]–[22]. In addition, orexin neurons contain other peptides including dynorphin, and neuronal activity-regulated pentraxin (Narp) [23]–[25]. These factors may play cooperative roles with orexin, although orexin is likely to play a central role in the function of these neurons, because *orexin* ^−/−^ mice and orexin neuron-ablated mice show almost the same phenotype [26].

Both neurotensin and orexin have been shown to localize in the LHA and activate dopaminergic neurons in the ventral tegmental area (VTA). A recent study showed that mice null for the leptin receptor (LepRb) specifically in neurotensin neurons showed early-onset obesity, modestly increased feeding, and decreased locomotor activity. This study also showed that neurotensin neurons send innervations to orexin neurons [27].

Here, we further studied the interaction of neurotensin and orexin and investigated the physiological relevance of this interaction in vivo. We found that many neurotensin-expressing neurons in the LHA also produce orexins. We also show that neurontensin plays an important role in maintenance of activity of orexin neurons and physiological regulation of sleep/wakefulness states.

## Results

### Neurotensin and orexin are co-localized in the lateral hypothalamic area

**Figure 1:**
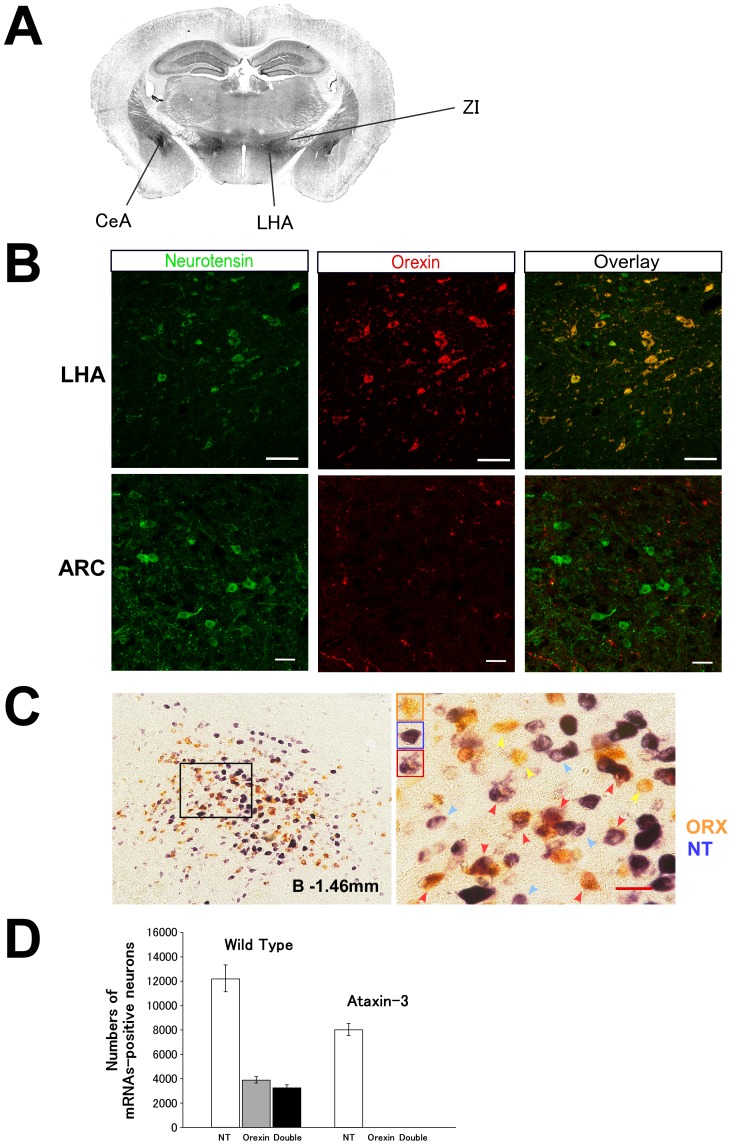
Neurotensin colocalizes with orexin in LHA neurons. A, Immunostaining of neurotensin in a coronal section of mouse brain. Dense staining of neurotensin-like immunoreactivity is observed in the central nucleus of the amygdala (CeA), lateral hypothalamic area (LHA) and zona incerta (ZI). B, Double immunofluorescence study showing that neurotensin-containing neurons also express orexin in the LHA but not in the arcuate nucleus (ARC). Upper panels, neurotensin (green)- and orexin (red)-like immunoreactivity in the LHA (upper panels) and arcuate nucleus (lower pannels). Scale bar indicates 20 µm. C, Double-label in situ hybridization histochemistry showing distribution of *orexin* (brown) and *neurotensin* (blue) mRNAs. Left panel, Representative low power image of the LHA region (bregma −1.46 mm) of a coronal section of C57BL/6 mouse brain. Right panel, higher power view of the region indicated by a black rectangle in the left panel. Yellow arrowheads show *prepro-orexin* mRNA, and blue arrowheads show *neurotensin* mRNA. Red arrowheads show neurons expressing both *neurotensin* and *orexin* mRNAs. Rectangles show typical examples of orexin-single-positive, neurotensin-single-positive, and double-positive cells. Scale bar indicates 20 µm. D, Numbers of neurotensin- or orexin-positive neurons in LHA of wild-type (Wild-Type, n = 5) and *orexin-ataxin-3* (Ataxin-3, n = 5) mice revealed by double labeling in situ hybridization. White, grey and black bars show neurotensin-, orexin- and double-positive neurons, respectively.

Immunostaining showed that many neurotensin-containing cells were observed in the LHA, zona incerta (ZI) and central nucleus of the amygdala (CeA) (Figure 1A). Orexins are also well known to be expressed in the LHA [10], [15], [28]. Although neurotensin-positive neurons were distributed more widely in the brain (Figure 1A, also see Allen Brain Atlas, Nts-Coronal-05-3215: Nts_260 (position: 6500)), with several discrete regions containing many positive cells, including the LHA, ZI, and CeA, the regions of distribution of orexin neurons and neurotensin neurons in the LHA partially overlapped.

We first examined the relationship between these neuropeptides in the LHA by dual-labeling immunofluorescent study (Figure 1B, upper panels). This study revealed that 82% of orexin neurons in the LHA also expressed neurotensin. We also observed the existence of neurotensin-positive neurons in the arcuate nucleus (ARC) in the same section. However, we did not find any co-localization of orexin in these cells, although we observed orexin-ir fibers in this region (Figure 1B, lower panels). This observation confirmed the specific labeling of orexin and neurotensin.

Double label in situ hybridization also confirmed the co-localization (Figure 1C D). This study showed that 84% of orexin neurons also expressed *Neurotensin* mRNA, being concordant with the result of double immunofluorescence. Likewise, 27% of neurotensin neurons in the LHA expressed *Orexin* mRNA.

By in situ hybridization, we also found that the number of *Neurotensin*-positive neurons in the LHA of *orexin/ataxin-3* mice, in which orexin neurons are specifically ablated [26], was markedly lower than that in wild- type mice with the same genetic background (C57BL/6J) (Figure 1D). This finding further supports that some neurotensin-containing neurons in the LHA are orexin neurons, which are specifically ablated in *orexin/ataxin-3* mice [26].

### Neurotensin activates orexin neurons

**Figure 2:**
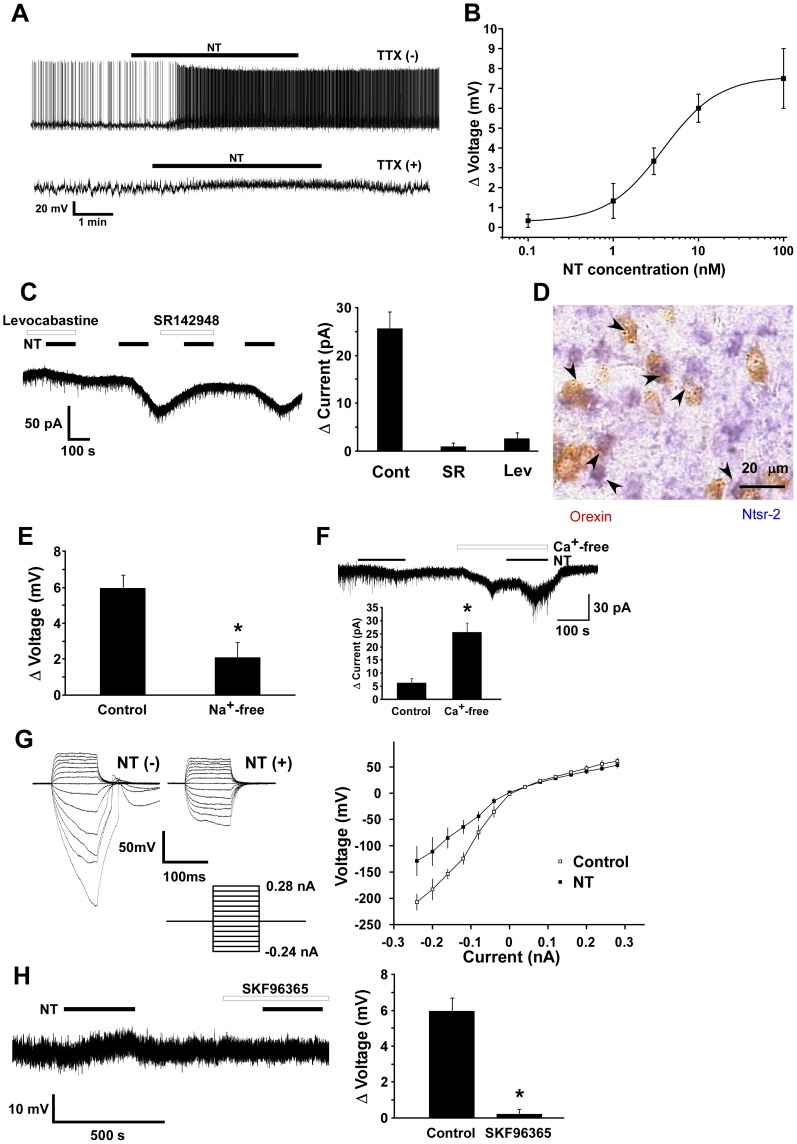
Neurotensin activates orexin neurons. **A**, Whole cell current clamp recording of orexin neurons shows that neurotensin (100 nM) depolarized orexin neurons in the absence (top) or presence (bottom) of TTX (1 µM). **B**, Concentration dependence of neurotensin-induced depolarization (mV) in the presence of TTX. EC_50_ and Emax were 3.84±0.02 nM and 7.60±0.01 mV, respectively (n = 3–5). **C**, Effects of NTSR-1 or NTSR-2 antagonist on effects of neurotensin on orexin neurons. Both SR142948 (n = 7, 10 µM), a non-selective antagonist, and levocabastine (n = 7, 1 µM), an NTSR-2 preferential antagonist, almost completely blocked the effects of neurotensin-induced inward current in orexin neurons. Left panel, a typical trace. Right panel, effect of neurotensin-induced current in orexin neurons in the presence of SR142948 or lebocavastine. Extracellular solution was used as vehicle control. **D**, Double-labeling in situ hybridization shows most neurons expressing *orexin* mRNA (red) also express *Ntsr-2* mRNA (blue). Black arrowheads show colocalization. Scale bar indicates 20 µm. **E**, Effect of extracellular Na^+^ on neurotensin-induced depolarization. NaCl was replaced by an equimolar concentration of choline chloride in the presence of TTX (1 µM) (Na^+^-free). Neurotensin (10 nM)-induced depolarization was markedly decreased in Na^+^-free solution (n = 4). **F**, Effect of extracellular Ca^2+^ on neurotensin-induced inward current. Neurotensin (10 nM) induced an inward current in the presence of extracellular calcium. This inward current was markedly increased in Ca^2+^-free extracellular solution (n = 7). Lower panel shows the fold-increase in inward current (pA) in Ca^2+^-free solution. **G**, Current-voltage relationship obtained by the current step protocol using a CsCl pipette in K^+^- and Ca^2+^-free extracellular solution. The steady state potential, at the end of the current step (marked by circle in left panel), is plotted in the current-voltage relationship in the right panel. I-V curve shows that the reversal potential of the neurotensin (10 nM)-induced current was 9.82 mV (n = 6–10). Neurons were current-clamped and the current was stepped from –240 pA to +280 pA (with increments of 40 pA with a duration of 100 ms). Open circle indicates control and filled circle indicates neurotensin (10 nM) application. **H**, Effects of non-selective cation channel blocker, SKF96365. Left panel, SKF96365 (10 µM) inhibited neurotensin-induced depolarization. Right panel, Bar graph indicates that SKF96365 inhibited neurotensin-induced depolarization (n = 4). Values are mean±S.E.M. *, p<0.05.

Recent studies have suggested that the activity of orexin neurons is influenced by various factors [8]. We previously found that neurotensin increases the intracellular calcium concentration ([Ca^2+^]_i_) of orexin neurons [19]. We analyzed the mechanisms by which neurotensin activates orexin neurons in detail by patch clamp electrophysiological studies using brain slice preparations from *orexin/EGFP* mice [29]. Under whole-cell current clamp mode, neurotensin (100 nM) bath application depolarized orexin neurons, with an increase in firing frequency (Figure 2A). Neurotensin (100 nM) significantly increased the firing frequency to 663 ± 243% (n = 6, p = 0.0123) of control. Depolarization was also observed in the presence of TTX (Figure 2A) (6.0±0.7 mV, n = 5), suggesting that neurotensin directly activates orexin neurons. The response peaked 40–80 sec after application of neurotensin, and the membrane potential returned to the basal level in 2–4 min after washout. The majority of GFP-positive neurons tested were depolarized by neurotensin (91%, 64 out of 70), and a few orexin neurons showed no response or slight depolarization (9%, 6 out of 70). On the other hand, 56% (10 out of 18) of GFP-negative neurons in the same area were depolarized by neurotensin and the rest showed no response to neurotensin. Figure 2B demonstrates that neurotensin depolarized orexin neurons in the presence of TTX in a concentration-dependent manner; EC_50_ and maximum effect (E_max_) were 3.84±0.02 nM and 7.60±0.01 mV, respectively (n = 3–5). At a holding potential of −60 mV under voltage clamp, neurotensin (10 nM) induced an inward current in orexin neurons in the presence of TTX (6.4±1.7 pA; n = 5) (Figure 2F).

### Neurotensin receptor-2 (NTSR-2) is involved in neurotensin-induced excitation of orexin neurons

To identify the subtypes of neurotensin receptors involved in neurotensin-induced excitation of orexin neurons, we used a non-selective neurotensin receptor antagonist, SR142948, and an NTSR-2 selective antagonist, levocabastine. Under voltage-clamp mode in calcium-free extracellular solution, at a holding potential of −40 mV, neurotensin (10 nM) induced 25.7±3.5 pA of inward current (n = 7) (Figure 2C). The neurotensin-induced inward current in orexin neurons was significantly and almost completely inhibited by SR142948 or levocabastine (Figure 2C). In the presence of 10 µM SR142948 or 1 µM levocabastine, the neurotensin-induced current was inhibited to 1.0±0.7 pA (n = 7, p<0.0001) or 2.7±1.2 pA (n = 7, p<0.0001), respectively (Figure 2C, right panel).

To histologically examine the expression of *Ntsr*-1 and *Ntsr*-2 in orexin neurons, we performed double in situ hybridization, using DIG-labeled antisense riboprobes for *Ntsr-1* or *Ntsr-2* mRNA together with FITC-labeled antisense riboprobes for *prepro-orexin* mRNA. This study revealed that *Ntsr-2*-expressing cells, presumably including astrocytes, exist diffusely in the CNS, consistent with previous observations [30], [31]. *Orexin* mRNA was colocalized with *Ntsr-2* mRNA in the LHA (Figure 2D). On the other hand, *Ntsr-1* was not detected in the LHA, although abundant expression of *Ntsr-1* was observed in the VTA (not shown). These results suggest that NTSR-2, a levocabastine-sensitive subtype of neurotensin receptor, is almost exclusively involved in neurotensin-induced activation of orexin neurons.

### Activation of non-selective cation channels is involved in neurotensin-induced activation of orexin neurons

To examine the mechanisms of the neurotensin-induced current in more detail, additional electrophysiological experiments were performed. First, the effect of extracellular Na^+^ in neurotensin-induced depolarization was studied. Experiments were performed in which NaCl in the extracellular solution was replaced by an equimolar concentration of choline chloride in the presence of TTX (1 µM). When choline was substituted for sodium, neurotensin-induced depolarization was strongly depressed. In Na^+^-free solution, neurotensin (10 nM)-induced depolarization was decreased from 6.0±0.8 mV (n = 4) to 2.1±0.8 mV (n = 4) (Figure 2E) (p = 0.0114), suggesting that neurotensin-induced depolarization is primarily dependent on extracellular Na^+^. Next, to examine the contribution of calcium ions in this response, calcium was removed from the extracellular solution. Removal of extracellular calcium markedly potentiated the neurotensin-induced inward current measured by voltage clamp recording holding at −60 mV (Figure 2F). In the presence and absence of calcium ions in the extracellular solution, the neurotensin (10 nM)-induced inward current was 6.4±1.7 pA (n = 5) and 25.7±3.5 pA (n = 7), respectively (p = 0.0015). Therefore, the neurotensin-induced inward current increased about 4 fold in calcium-free solution, suggesting that it was physiologically suppressed by extracellular calcium ions. The reversal potential of the neurotensin-induced current in potassium-free and calcium-free extracellular solution (in mM: 140 NaCl, 2 CsCl, 1 MgCl_2_, 1 EGTA, 10 HEPES, 10 glucose) was near 0 mV (−6.4 mV, n = 6–10) when measured using a CsCl pipette solution (in mM: 145 CsCl, 1 MgCl_2_, 10 HEPES, 1.1 EGTA, 0.5 Na_2_GTP, and 2 MgATP) (Figure 2G). This reversal potential is consistent with activation of nonselective cation channels. Several recent reports suggest that the transient receptor potential (TRP) channel family plays an important role in the receptor-operated influx of cations [32], [33]. In addition, the current through TRP channels is known to be suppressed by the presence of extracellular calcium ions, as with neurotensin-induced inward currents (Figure 2F)[34], [35]. Collectively, our observations suggest that TRP channels are involved in neurotensin-induced activation of orexin neurons. To evaluate this possibility, the effect of SKF96365, which is often used as a TRP channel blocker [36], was tested on neurotensin-induced depolarization of orexin neurons. SKF96365 significantly inhibited neurotensin-induced depolarization (Figure 2H). In the presence and absence of SKF96365 (10 µM), neurotensin-induced depolarization was 0.25±0.25 mV (n = 4) and 6.00±0.71 mV (n = 5), respectively (Figure 2H) (p = 0.0002). This observation further supports the idea that neurotensin activates orexin neurons through TRP channels.

### Effects of neurotensin antagonists on activity of orexin neurons *in vivo*

**Figure 3:**
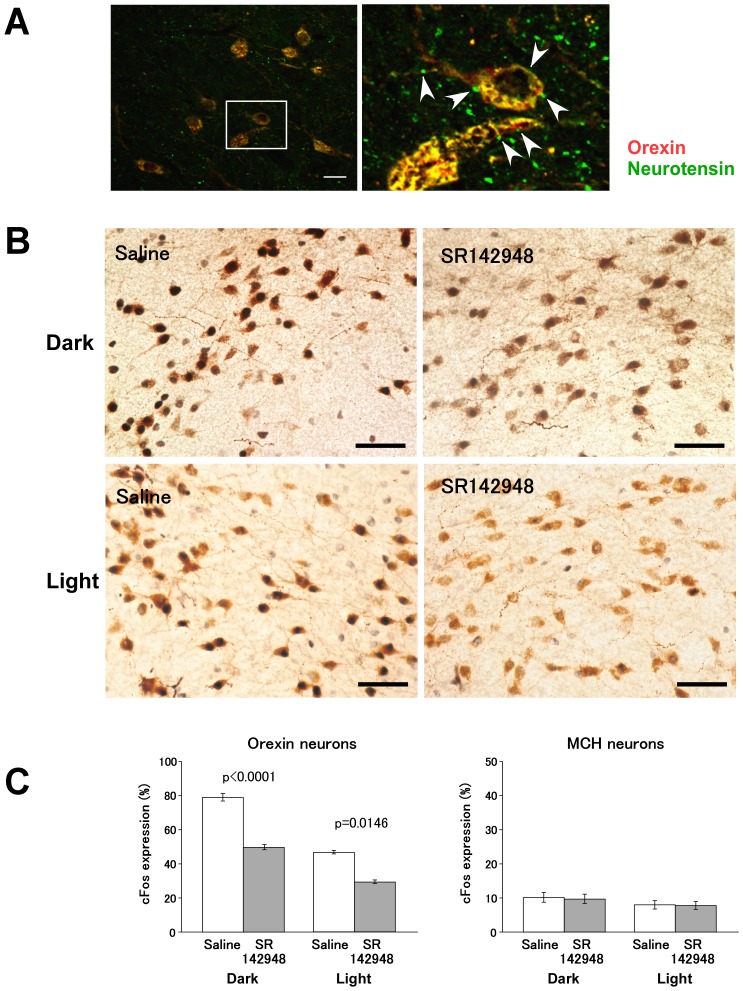
Neurotensinergic fibers appose orexin neurons. **A**, Double immunofluorescence study shows that many varicose terminals with neurotensin-like immunoreactivity (green) make appositions to orexin neurons (red) in the LHA (Left panels). Right panel, high power view of rectangular region in left panel. **B**, Photomicrograph showing distribution of Fos (black nuclear label) and orexin (brown label) expression in LHA. Scale bar indicates 40 µm. Upper panels, left panel, control (saline injection); right panel, SR142948 injection at 20:45. Animals were sacrificed at 21:45 and subjected to immunostaining. Lower panels, left panel, control (saline injection); right panel, SR142948 injection at 8:45. Animals were sacrificed at 9:45 and subjected to immunostaining. **C**, SR142948 administration decreased Fos immunoreactivity of orexin neurons, but not in MCH neurons.

We found that neurotensin-immunoreactive fibers make appositions to orexin neurons by immunohistochemical studies (Figure 3A). To decipher the physiological relevance of neurotensin in maintaining the activity of orexin neurons in vivo, we intraperitoneally administered a non-selective specific neurotensin antagonist SR142948 in male mice at 8:45 and examined the activity of orexin neurons by means of Fos immunostaining at 9:45. We found that SR142948 (1 mg/kg) administration significantly decreased the number of orexin neurons expressing Fos immunoreactivity (Control, 46.60±5.42% (n = 6);SR142948, 29.25±2.31% (n = 6), p = 0.0146) (Figure 3B, C). In contrast, the number of MCH neurons expressing Fos immunoreactivity in the same area was not affected by SR142948 (Control, 8.03±1.23% (n = 6), SR142948, 7.84±1.14% (n = 6), p = 0.9113) (Figure 3C). The decrease of Fos expression in orexin neurons was also observed when SR142948 was administered at 20:45 and examined at 21:45 (Control, 79.00±2.21% (n = 5); SR142948, 49.80±1.51% (n = 5), p<0.0001) (Figure 3C). We obtained similar result when SR142948 was administered the third ventricle at 10:00 (Figure S1). These observations support the idea that neurotensin maintains the activity of orexin neurons but not MCH neurons in vivo.

We also examined the effect of SR142948 on behavioral level. We examined the effect of SR142948 on sleep/wakefulness states in mice. SR142948 (1 mg/kg or 3 mg/kg), dissolved in dimethyl sulfoxide (DMSO) and diluted in saline to a final concentration of 1% DMSO, was administered intraperitoneally to mice at the start of the light phase (08:45) or dark phase (20:45). Control injection was performed with saline containing the same concentration of DMSO as with injection of SR142948. EEG and EMG were recorded for four hours to score sleep/wakefulness states.

**Figure 4:**
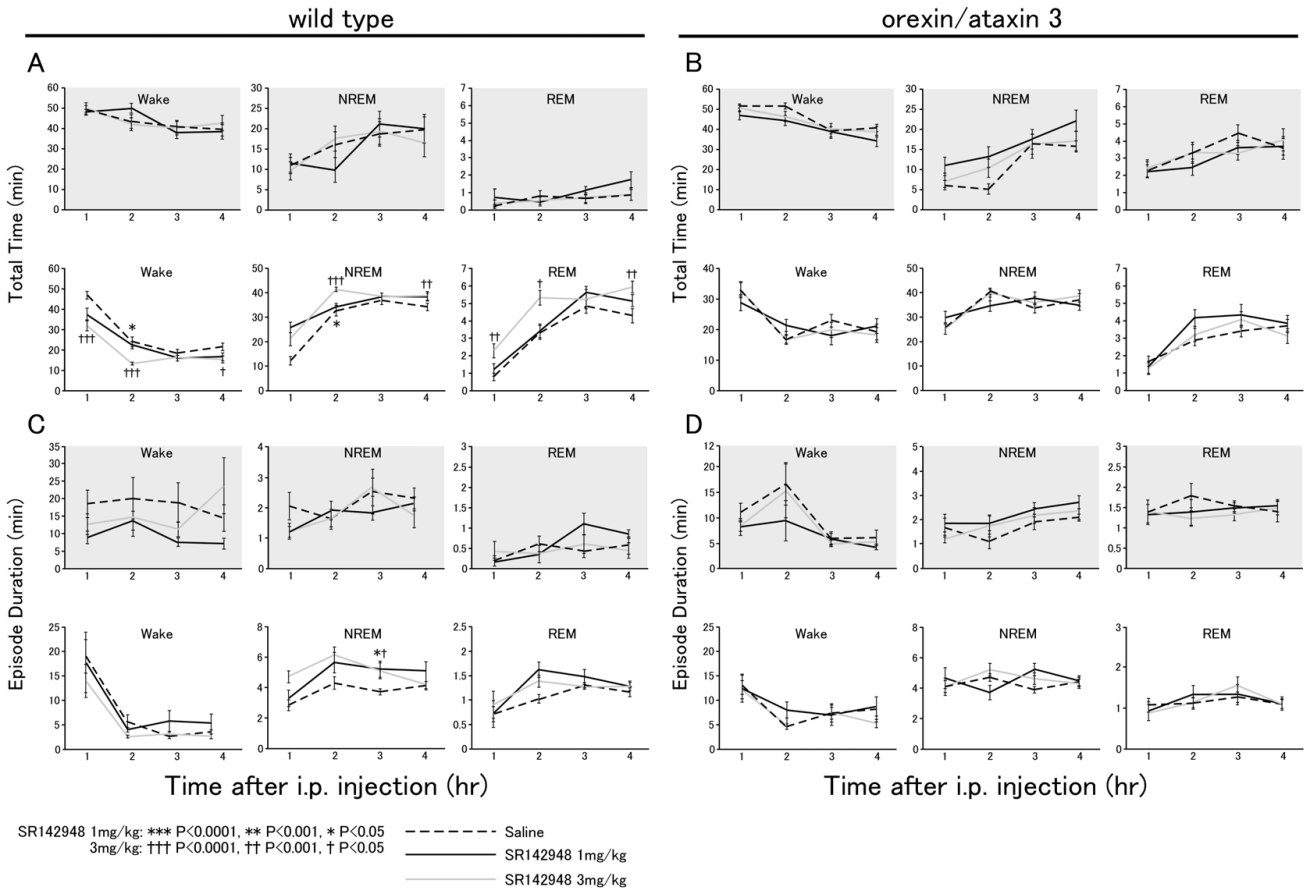
A neurotensin receptor antagonist SR142948 dose-dependently decreased wakefulness time and duration during the lights on period in wild-type mice, but not in orexin neuron-ablated mice. Hourly amounts (A, B) and average episode duration (C, D) of awake, non-REM, and REM sleep states (mean±SE) plotted over 4 hr after administration of saline (dotted line) or SR142948 (solid line) in wild-type (n = 27, 14 and 7 for saline, SR 1 mg/kg, and 3 mg/kg, respectively) (A, C) and *orexin/ataxin-3* mice (n = 14, 14 and 7 for saline, SR 1 mg/kg, and 3 mg/kg, respectively) (B, D). Data for the dark phase are displayed in shaded panels. SR142948 was administered at the start of light or dark periods (8:45 or 20:45).

Wild-type mice injected with SR142948 showed a significantly shorter wakefulness time and longer NREM and REM sleep times as compared with mice with vehicle injection for 2 hour after administration at the start of the light period (Figure 4A, lower panels). This effect was dose-dependent, and accompanied by lengthening of both NREM and REM sleep time (Figure 4A, lower panels). Fast Fourier transform (FFT) revealed that the microarchitecture of the EEG spectra during the non-REM phase of saline- and SR-injected groups two hours after injection were identical in both wildtype and *orexin-ataxin 3* mice, suggesting the antagonist induced normal NREM sleep (r = 0.999281 and 0.99577 for saline vs SR 1 mg and 3 mg, respectively in wild type mice, r = 0.987997 and 0.999455 for saline vs SR 1 mg and 3 mg, respectively in orexin-ataxin 3 mice) (Figure S3).

To examine whether this effect of SR142948 is caused by inhibition of orexin neuronal activity, we also administered SR142948 to *orexin/ataxin-3* mice in which orexin neurons are genetically ablated [26]. SR142948 failed to influence any sleep parameters in the *orexin/ataxin-3* transgenic mice (Figure 4B, D). We did not find any effects of SR142948 on sleep parameters in both wild-type and *orexin/ataxin-3* mice in the dark period. This result is consistent with a recent report showing that optogenetic inhibition of orexin neurons induces NREM sleep in the light period, but not in the dark period [37]. Orexin in CSF are known to show circadian variation, high at the end of active period[38]. In order to examine the possibility that abundant orexin in the extracellular space eliminated the effect of neurotensin blocker, we also performed the experiment at the end of dark period (SR142948 was administered 2hr before the end of dark period). We again failed to find effect of SR142948 on sleep parameters in wild type or *orexin/ataxin-3 mice* (Figure S2). This observation further suggested a possibility that the blockade of neurotensin does not evoke the effect on the activity of orexin neurons during the level of endogenous orexin is high [39].

These observations suggest that neurotensin physiologically maintains activity of orexin neurons, and inhibition of the neurotensinergic system affects the activity of orexin neurons to increase NREM sleep in the light period.

## Discussion

### Neurotensin is involved in autoactivation of orexin neurons

LHA neurons express a variety of neuropeptides, including orexin, melanin-concentrating hormone, QRFP and neurotensin [9], [27], [40], [41]. Among them, both neurotensin and orexin have been implicated in feeding and reward systems, and shown to activate dopaminergic neurons in the VTA [42]. Functional interaction between these peptides might have an important physiological role.

The present study showed that orexin neurons in the LHA co-expressed neurotensin (Figure 1B, C). The finding that the number of neurotensin neurons in the LHA was decreased in *orexin/ataxin-3* mice further suggests the co-localization of neurotensin and orexin (Figure 1D). In addition, neurotensin activated most of the orexin neurons (64 of 70). Orexin neurons are reported to be innervated by orexin-immunoreactive fibers and activated by orexins through orexin 2 receptors [39]. Recently, it was shown that neurotensin-immunoreactive fibers also make appositions to orexin neurons [27]. We also found that neurotensin-immunoreactive fibers made appositions to orexin neurons (Figure 3A). These findings suggest a possibility that neurotensin released from orexin neurons also acts as an auto-stimulator of orexin neurons. Some of these neurotensin-immunoreactive fibers are likely to come from neurotensin-containing neurons in the LHA, which also express orexin, suggesting that neurotensin is involved in autoregulation of orexin neuronal activity. Neurotensinergic neurons in the preoptic area, bed nucleus of the stria terminals, central nucleus of the amygdala, and the VTA are other possible sources for these innervations, because these regions were reported to contain neurotensinergic neurons [43] and to innervate orexin neurons [16], [17].

These findings suggest that neurotensin is colocalized in some orexin neurons and is involved in auto-regulation of these neurons through autocrine and/or paracrine mechanisms. We found that neurotensin-positive neurons in the LHA of orexin neuron-ablated, orexin/ataxin-3 mice were markedly decreased as compared with wild type mice (Figure 1D). A recent study showed that mice null for the leptin receptor (LepRb) specifically in neurotensin neurons showed early-onset obesity, modestly increased feeding, and decreased locomotor activity [27]. These observations raise a possibility that the late onset obesity seen in orexin-ataxin 3 mice might partly stem from the decreased basal neurotensin level in these mice.

### Neurotensin activates orexin neurons through NTSR-2 and non-selective cation channels

Neurotensin-induced depolarization and the inward current were not affected by TTX, suggesting a direct postsynaptic effect. Studies using selective antagonists suggested that NTSR-2 is involved in neurotensin-induced activation of orexin neurons, because a NTSR-2 antagonist, levocabastine, almost completely abolished the response. Additionally, in situ hybridization histochemistry revealed that most of the orexin neurons expressed *Ntsr-2* mRNA (Figure 2D). We also found that neurotensin activated non-selective cation channels in orexin neurons (Figure 2E-H), which are similar to the channels activated by CCK-8S [19], vasopressin and oxytocin [22]. The inward current induced by neurotensin was strongly potentiated by the removal of extracellular calcium ions and was inhibited by SKF96365. These characteristics are similar to those of nonselective cation channels activated by CCK-8S, vasopressin and oxytocin in orexin neurons, suggesting that the same non-selective cation channels might be involved in the neurotensin-induced response. TRP-C channels are a possible candidate for such channels because they have been shown to be activated by diacyl glycerol produced by the phospholipase C pathway [44], which is downstream of Gq-coupled receptors such as Ntsr-2. The current through TRP channels is known to be increased by the removal of extracellular calcium ions[34], [35], further supporting the possibility of the involvement of this type of channel in the response.

### Physiological relevance of neurotensin-mediated activation of orexin neurons

We hypothesized that the basal activity of orexin neurons might be at least partially maintained by neurotensin. To evaluate this possibility, we administered a non-selective neurotensin receptor antagonist, SR142948, to mice, and examined the number of orexin neurons that showed Fos-like immunoreactivity in their nuclei. SR142948 significantly decreased the number of Fos-positive orexin neurons in both dark and light periods (Figure 3B, C). Next, to examine the behavioral effects induced by the decrease in activity of orexin neurons by SR142948, we examined the effect of SR142948 on sleep/wakefulness states in mice. We observed that SR142948 decreased wakefulness time accompanied by increased NREM and REM sleep times in wild-type mice for two hours when administered at the start of the light period (Figure 4). However, it failed to elicit any effect on sleep/wakefulness parameters in *orexin/ataxin-3* transgenic mice (Figure 4B, D). Although wakefulness time in *orexin/ataxin-3* mice was comparable to that in wild-type mice, which is consistent with our previous data [26], SR142948 did not elicit effects on sleep/wakefulness states in *orexin/ataxin-3* mice, suggesting that effects of neurotensin on the activity of orexin neurons play an important role in sleep/wake regulation (Figure 4). These observations suggest that neurotensin affects sleep/wakefulness states through regulating the activity of orexin neurons.

We observed a longer wakefulness time after injection of saline at the start of the light period as compared with SR142948 injection or *orexin/ataxin-3* mice with both saline and SR142948 injection. This might suggest that stress induced by the injection procedure increased wakefulness time in wild-type mice with saline injection through excitation of orexin neurons, and this effect was blocked by SR142948.

We did not find any obvious effect of SR142948 on sleep/wakefulness behavior in wild-type mice when administered at the start of the dark period. This is consistent with a report that showed that acute optogenetic silencing of orexin neurons decreased wakefulness time during the light period, but not during the dark period [37]. This might suggest that because the firing rate of orexin neurons is high in the dark period, and released orexin peptide has a long half-life in the extracellular space, acute silencing of orexin neurons failed to affect behavioral state. Administration of SR142948 two hours before the end of dark period, when CSF orexin level is high, also failed to evoke any effect on sleep parameters in wild type mice (Figure S2), further supporting this idea. However, during the light period, since the firing rate of orexin neurons is relatively low, and only a small amount of orexin peptide is released, inhibition of orexin neurons might efficiently affect the behavioral state. Alternatively, the arousal-regulated region might be already sufficiently activated without the help of the orexin system during the dark period; therefore, silencing of orexin neurons might fail to affect the behavior of mice.

Our present study suggested that neurotensin colocalizes with orexin and plays an important role in maintaining the activity of orexin neurons. Recently, it was shown that orexin neurons are activated by orexin itself [39]. These two mechanisms might co-operatively and redundantly maintain the firing rate of orexin neurons.

*Ntsr-2-*expressing cells, including astrocytes, exist diffusely in the CNS [2], [45]. Therefore, to examine the precise role of neurotensin-mediated regulation of orexin neurons, mice with spatially-restricted disruption of *Neurotensin* or *Ntsr-2* in orexin neurons would be required. Such a genetic model would further reveal the physiological relevance of the neurotensin-orexin system, including its role in the regulation of dopaminergic neurons.

In conclusion, neurotensin colocalizes with orexin and activates orexin neurons. This activation involves NTSR-2-mediated activation of TRP channels. These influences of neurotensin on orexin neurons are necessary to maintain the activity of orexin neurons in vivo, and might have an important role in the autoregulatory function of the orexin system.

## Experimental Procedures

### Drugs

Neurotensin (Peptide Institute, Osaka, Japan), SKF96365 (Sigma, St. Louis, MO), CNQX, AP-5 (Wako, Osaka, Japan), picrotoxin, tetrodotoxin (TTX) (Wako), SR142948 (TOCRIS Bioscience, Bristol, UK), pyrilamine (Sigma) and levocabastine (Sigma) were dissolved in extracellular solution.

### Animals

All experimental procedures involving animals were approved by the Animal Experiment and Use Committee of Kanazawa University (AP-101567) and were in accordance with NIH guidelines. All efforts were made to minimize animal suffering and discomfort and to reduce the number of animals used. Genetically-modified mice used in this study were *orexin/EGFP* mice [46] and *orexin/ataxin-3* mice [26]. These mice were crossed to wild type C57BL/6J mice for more than seven generations. Wild-type littermates of these transgenic mice were used as the wild-type controls. Mice were maintained under a strict 12 hour light:dark cycle (light on at 8:45 a.m., off at 8:45 p.m.) in a temperature (22°C) and humidity controlled room and fed ad libitum.

### *In situ* hybridization

Double in situ hybridization was performed according to procedures previously described [47]. Digoxigenin (DIG)-labeled riboprobes for *Neurotensin* were synthesized from a 510 bp fragment of murine *Neurotensin* cDNA (nucleotides −96 − +605 from the initiation codon) containing 510 bp of the whole coding region cloned into pCRII vector (Invitrogen). Fluorescein (FITC)-labeled riboprobes for *prepro-orexin* were synthesized by in vitro transcription with pBluescript II SK (+) containing a 0.5 kb fragment encoding Gln33-Val130 of rat *prepro-orexin* [7]. Digoxigenin (DIG)-labeled riboprobes for *mNtsr-1* were synthesized from a 0.86 kb fragment of *mNtsr-1* cDNA (nucleotides −233 − +627 from the initiation codon) containing 0.23 kb of the 5′-noncoding region and the 5′-most 0.63 kb of the coding region cloned into pBluescript IISK (+). Full-length cDNA for *mNtsr-2* cloned in pBluescript II SK (-) was donated by Dr. Wada. Digoxigenin (DIG)-labeled riboprobes for *mNtsr-2* were synthesized by in vitro transcription using this construct. The DIG and FITC-labeled probes were detected by anti-DIG (1/2000) and anti-FITC (1/1000) antibodies conjugated with alkaline phosphatase (Roche Diagnostics, Basel, Switzerland). Alkaline phosphatase activity was detected with NBT/BCIP and INT/BCIP (Roche Diagnostics).

### Immunohistochemistry

Mice were anesthetized deeply with diethyl ether and perfused sequentially with 20 ml ice-cold saline and 20 ml cold 4% paraformaldehyde in 0.1 M phosphate buffer. The brains were removed and immersed in the same fixative solution for 24 hr at 4°C, and then immersed in 30% sucrose solution for at least 2 days. The brains were quickly frozen in embedding solution (Sakura Finetechnical Co. Ltd., Tokyo, Japan) and cut into coronal sections using a cryostat (HM 505E, Microm, Walldorf, Germany). To detect orexin and neurotensin immunoreactivity, coronal brain sections of C57BL/6 mice were incubated with goat anti-orexin antibody (1∶1000, Santa Cruz Biotechnology, Inc. Santa Cruz, CA, USA) at 4°C overnight, followed by incubation with Alexa Fluor 546-conjugated donkey anti-goat IgG (1∶400, Invitrogen Corp., Carlsbad, CA, USA) for 1.5 h at RT. Next, the same sections were incubated with rabbit anti-neurotensin antibody (1∶10,000, Merck KGaA, Darmstadt, Germany) at 4°C overnight. After washing with PBS, the sections were incubated with Alexa Fluor 488-labeled goat anti-rabbit IgG (1∶400, Invitrogen Corp.) for 1.5 h at RT. Double immunolabeling was detected with the aid of a fluorescence microscope (Zeiss Z1, Carl Zeiss AG, Göttingen, Germany) fitted with an optical-sectioning system (Zeiss Apotome, Carl Zeiss AG). To detect Fos immunoreactivity in orexin-expressing neurons, the sections were incubated with guinea pig anti-orexin antibody (Molecular Probes, 1∶1000; brown color) and rabbit anti-cFos antibody Ab-5 (Calbiochem, 1∶20000; black color).

### Quantitative analysis of immunolabeling

Cell counting was performed to determine numbers of orexin neurons, neurotensin neurons and Fos-expressing neurons. All orexin-positive, MCH-positive and neurotensin-positive neurons were counted bilaterally in the LHA, regardless of the intensity of their staining. For Fos counting, cells were considered Fos positive if their nucleus contained detectable black immunolabeling, regardless of the labeling intensity, and Fos negative if they displayed no visible nucleus or a nucleus lacking Fos immunolabeling. To obtain accurate estimates of cell number in sections, we used the three-dimensional counting method as described [48].

### Electrophysiological recording

*Orexin/EGFP* mice (2–6 weeks old) were anesthetized with isoflurane (Abbott, Osaka, Japan). The mice were decapitated under deep anesthesia. Brains were isolated in ice-cold cutting solution consisting of (mM): 280 sucrose, 2 KCl, 10 HEPES, 0.5 CaCl_2_, 10 MgCl_2_, 10 glucose, pH 7.4, bubbled with 100% O_2_. Brains were cut coronally into 300-µm slices with a vibratome (VTA-1000S, Leica, Germany). Slices containing the LHA were transferred for 1 hr to an incubation chamber at room temperature filled with physiological solution containing (mM): 140 NaCl, 2 KCl, 1 CaCl_2_, 1 MgCl_2_, 10 HEPES, 10 glucose, pH 7.4 with NaOH. These slices were transferred to a recording chamber (RC-27L, Warner Instrument Corp., CT, USA) at RT on a fluorescence microscope stage (BX51WI, Olympus, Tokyo, Japan). The fluorescence microscope was equipped with an infrared camera (C-3077 78, Hamamatsu Photonics, Hamamatsu, Japan) for infrared differential interference contrast (IR-DIC) imaging and a CCD camera (JK-TU53H, Olympus) for fluorescent imaging. Neurons that showed GFP fluorescence were subjected to patch-clamp recordings. Recordings were carried out with an Axopatch 200B amplifier (Axon Instruments, Foster City, CA) using a borosilicate pipette (GC150-10, Harvard Apparatus, Holliston, MA) prepared using a micropipette puller (P-97, Sutter Instruments, Pangbourne, UK) and filled with intracellular solution (4–10 MΩ) consisting of (mM): 125 K-gluconate, 5 KCl, 1 MgCl_2_, 10 HEPES, 1.1 EGTA-Na_3_, 5 MgATP, 0.5 Na_2_GTP, pH 7.3 with KOH. Osmolarity of the solution was checked with a vapor pressure osmometer (model 5520, Wescor, Logan, UT). The osmolarity of the internal and external solutions was 280–290 and 320–330 mOsm/l, respectively. The liquid junction potential of the patch pipette and perfused extracellular solution was estimated to be −16.2 mV and was applied to the data. The recording pipette was under positive pressure while it was advanced toward individual cells in the slice. Tight seals of 0.5–1.0 GΩ were made by negative pressure. The membrane patch was then ruptured by suction. The series resistance during recording was 10–25 MΩ and was compensated. The reference electrode was an Ag-AgCl pellet immersed in bath solution. During recordings, cells were superfused with extracellular solution at a rate of 1.0–2.0 ml/min using a peristaltic pump (Minipuls3, Gilson, Paris, France) at RT. Depolarizing and hyperpolarizing current pulses were applied to cells for durations of 200 msec at 20 pA steps at 2-sec intervals from the resting membrane potential (−60 mV) set by varying the intensity of a constantly injected current.

### Intracerebroventricular administration

Male mice (8 to 10 weeks old) were anesthetized with sodium pentobarbital (0.5 mg/kg, i.p.), positioned in a stereotaxic frame (David Kopf Instruments), and a guide cannula was implanted in the lateral ventricle under sterile conditions. The coordinates used to map the correct positioning of the implants were 0.2 mm posterior to the bregma, 0 mm lateral to the midline, and 5 mm below the skull surface. Mice were then housed singly for a recovery period of at least 7 days. SR142948 was dissolved in physiological saline, and delivered at a volume of 3 µl over 60 sec, and the injector was left in position for a further 60 sec to ensure complete dispersal. Administration was performed during the light period (10:00AM).

### Sleep recordings

An electrode for EEG and EMG recording was implanted in the skull of each mouse. The three arms of the electrode for EEG recording were placed approximately 2 mm anterior and 2 mm to the right, 2 mm posterior and 2 mm to the right, and 2 mm posterior and 2 mm to the left of the bregma. Stainless steel wires for EMG recording were sutured to the neck muscles of each mouse bilaterally, and each electrode was glued solidly to the skull. After the recovery period, animals were moved to a recording cage placed in an electrically shielded and sound attenuated room. A cable for signal output was connected to the implanted electrode and animals were allowed to move freely. Signals were amplified through an amplifier (AB-611J, Nihon Koden, Tokyo) and digitally recorded on a computer using EEG/EMG recording software (Vital recorder, Kissei Comtec). Animals were allowed at least seven days to adapt to the recording conditions prior to any EEG/EMG recording session. Following the adaptation period, each animal was intraperitoneally administered both SR142948 and saline on separate experimental days with a 3-day interval. The order of injection (i.e. either SR142948 first or saline first) was randomized. EEG/EMG data were evaluated and staged for 4 hours after administration. Data acquired on the day of saline administration were used as control. We analyzed FFT spectra of NREM period in 1-2hr epoch of saline- or SR-injected mice (N = 17). Power spectral analysis of EEG signals was performed using custom FFT software.

### Statistical analysis

Data were analyzed by two-way ANOVA followed by post hoc analysis of significance by Fisher's protected least significant difference test using the Stat View 5.0 software package for Macintosh (Abacus Concepts, Berkeley, CA, USA). Probability (*p*) values less than 0.05 were considered statistically significant.

## Supporting Information

Figure S1 Effect of icv administration of SR142948 on Fos expression in orexin neurons. A, Photomicrograph showing distribution of Fos (black nuclear label) and orexin (brown label) expression in LHA. Scale bar indicates 40 µm. Left panel, control (saline injection); right panel, SR142948 (5.0 nmol) injection at 10:00. Animals were sacrificed at 13:00 and subjected to immunostaining. B, SR142948 administration decreased Fos immunoreactivity of orexin neurons in a concentration-dependent manner (Control, 37.27±3.95%, n = 6; SR142948 (0.5 nmol), 31.28±5.11%, n = 6, p = 0.3762; SR142948 (5.0 nmol), 23.10±2.22%, n = 6, p = 0.0108, Student's t test) (left panel), but MCH neurons showed no response to SR 142948 (right panel).
(TIF)

Figure S2 Effect of a neurotensin receptor antagonist SR142948 on sleep parameters in wild-type mice and orexin/ataxin-3 mice when administered at two hours before the end of dark period. Hourly amounts (A, B) and average episode duration (C, D) of awake, non-REM, and REM sleep states (mean±SE) plotted over 4 hr after administration of saline (dotted line) or SR142948 (solid line) in wild-type (n = 14) (A, C) and orexin/ataxin-3 mice (n = 14) (B, D). Data for the dark phase are displayed in shaded panels.
(TIF)

Figure S3 Fast Fourier transform (FFT) revealed that the microarchitecture of the EEG spectra during the non-REM phase of saline- and SR-injected groups two hours after injection at the start of light phase are identical.
(TIF)
