# Supplementary figures and images for: Neurotensin Co-Expressed in Orexin-Producing Neurons in the Lateral Hypothalamus Plays an Important Role in Regulation of Sleep/Wakefulness States
Source: PLoS One. 2013 Apr 19;8(4):e62391. doi: 10.1371/journal.pone.0062391 (PMC3631195; doi:10.1371/journal.pone.0062391)

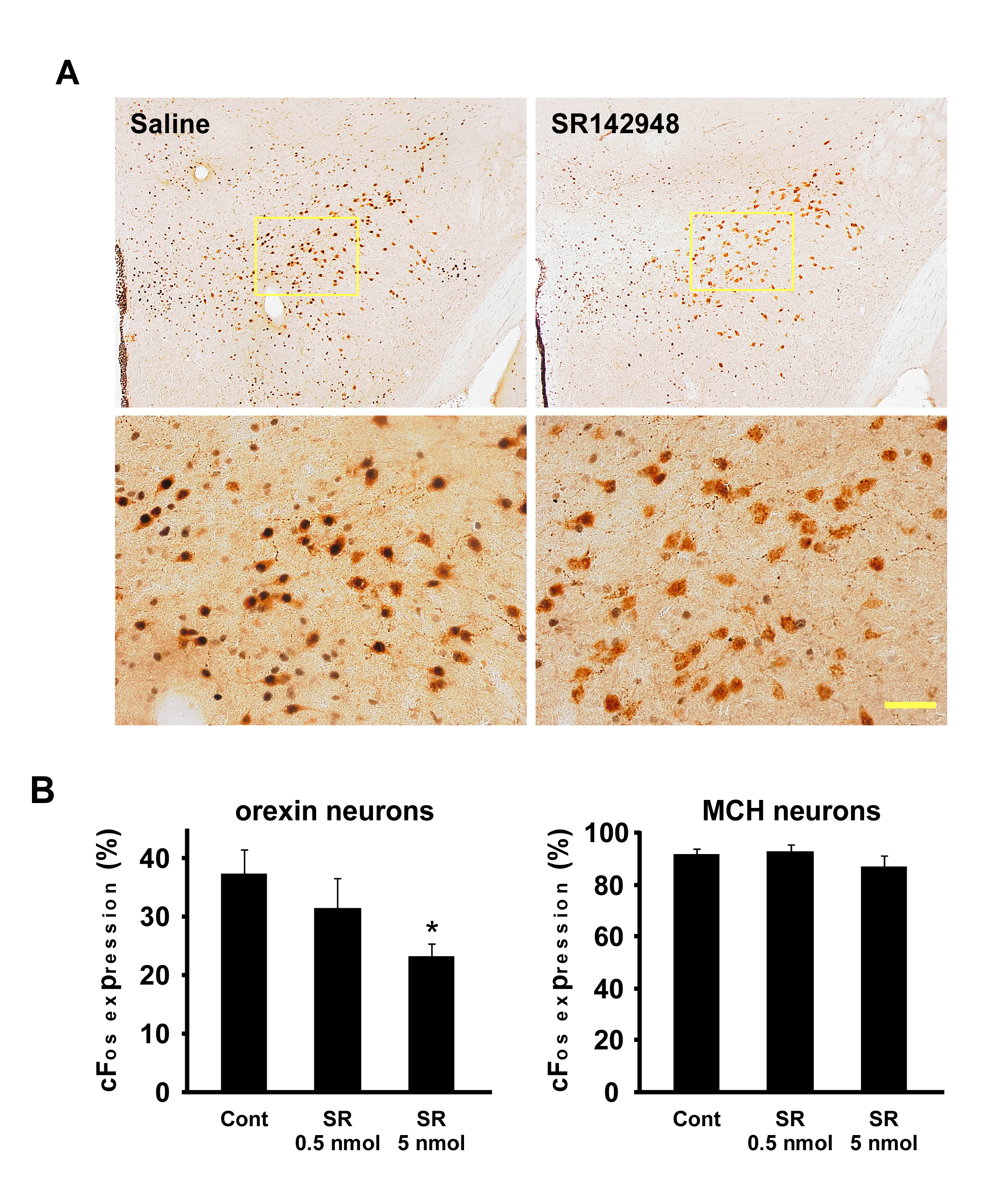

Supplement: Figure S1 — Effect of icv administration of SR142948 on Fos expression in orexin neurons. A, Photomicrograph showing distribution of Fos (black nuclear label) and orexin (brown label) expression in LHA. Scale bar indicates 40 µm. Left panel, control (saline injection); right panel, SR142948 (5.0 nmol) injection at 10:00. Animals were sacrificed at 13:00 and subjected to immunostaining. B, SR142948 administration decreased Fos immunoreactivity of orexin neurons in a concentration-dependent manner (Control, 37.27±3.95%, n = 6; SR142948 (0.5 nmol), 31.28±5.11%, n = 6, p = 0.3762; SR142948 (5.0 nmol), 23.10±2.22%, n = 6, p = 0.0108, Student's t test) (left panel), but MCH neurons showed no response to SR 142948 (right panel). (TIF) [file pone.0062391.s001.tif]

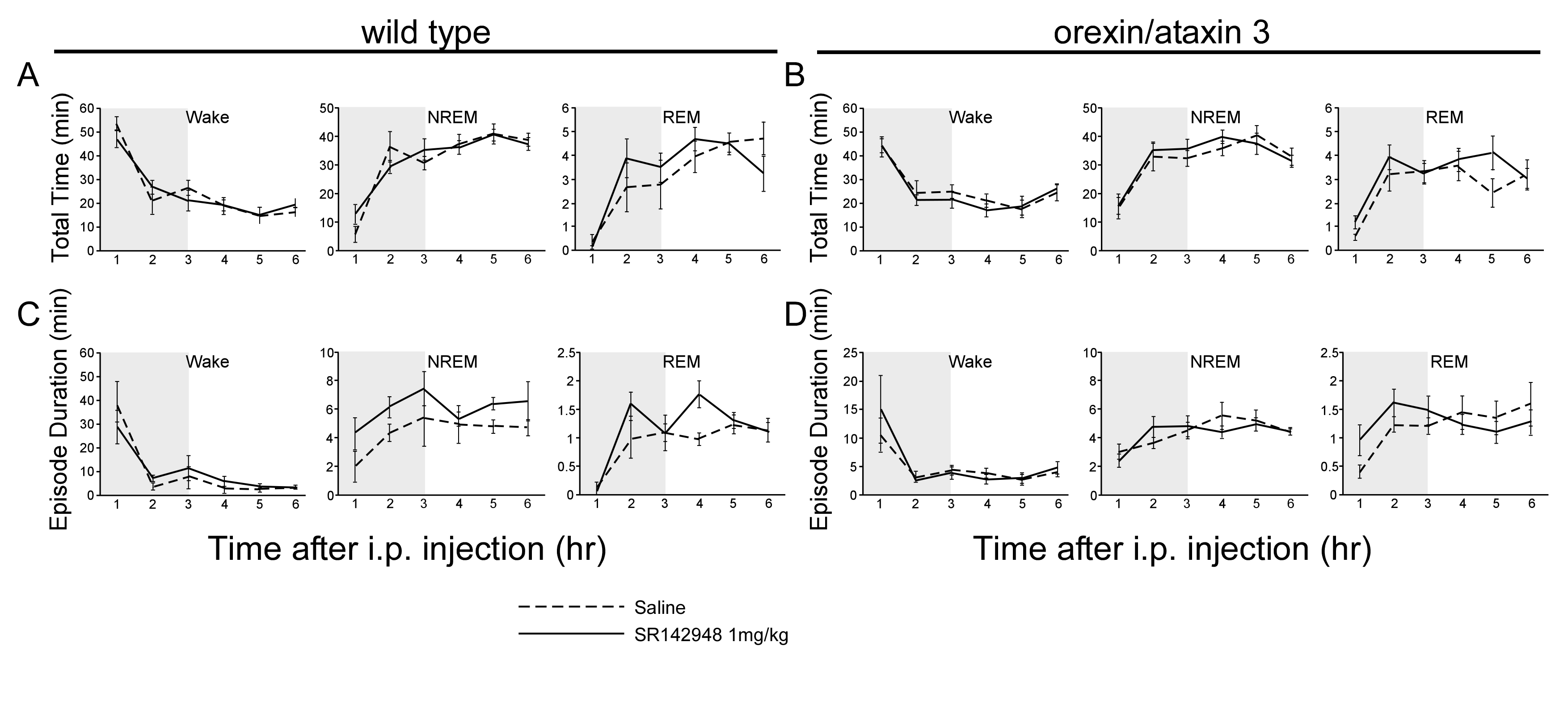

Supplement: Figure S2 — Effect of a neurotensin receptor antagonist SR142948 on sleep parameters in wild-type mice and orexin/ataxin-3 mice when administered at two hours before the end of dark period. Hourly amounts (A, B) and average episode duration (C, D) of awake, non-REM, and REM sleep states (mean±SE) plotted over 4 hr after administration of saline (dotted line) or SR142948 (solid line) in wild-type (n = 14) (A, C) and orexin/ataxin-3 mice (n = 14) (B, D). Data for the dark phase are displayed in shaded panels. (TIF) [file pone.0062391.s002.tif]

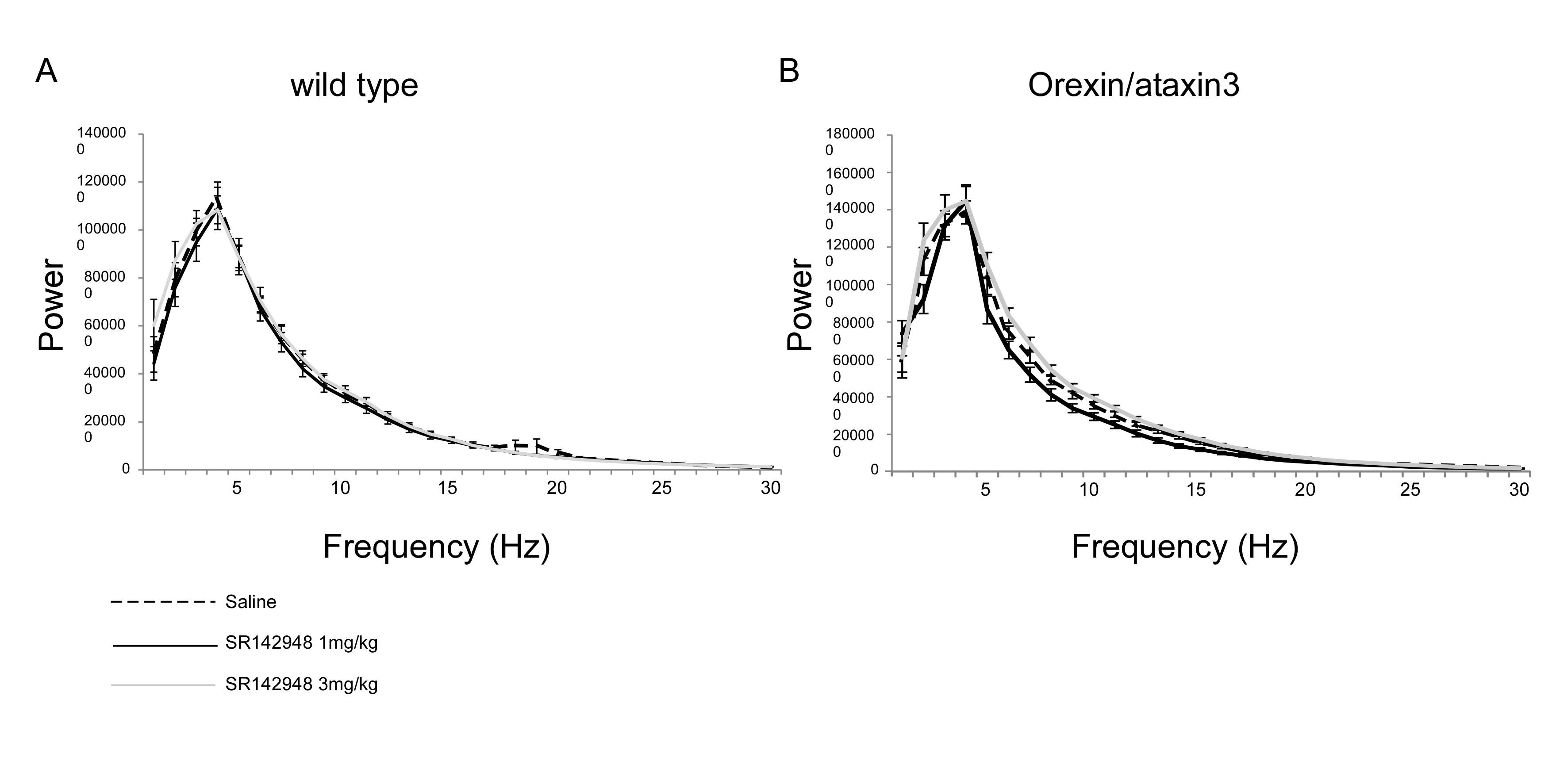

Supplement: Figure S3 — Fast Fourier transform (FFT) revealed that the microarchitecture of the EEG spectra during the non-REM phase of saline- and SR-injected groups two hours after injection at the start of light phase are identical. (TIF) [file pone.0062391.s003.tif]
